# Characterization of microRNA Expression Profiles of Murine Female Genital Tracts Following *Nippostrongylus brasiliensis* and Herpes Simplex Virus Type 2 Co-Infection

**DOI:** 10.3390/microorganisms13081734

**Published:** 2025-07-24

**Authors:** Roxanne Pillay, Pragalathan Naidoo, Zilungile L. Mkhize-Kwitshana

**Affiliations:** 1 Department of Biomedical Sciences, Faculty of Applied and Health Sciences, Mangosuthu University of Technology, Umlazi, Durban 4031, South Africa; thungaveloo.roxanne@mut.ac.za; 2 Department of Medical Microbiology, School of Laboratory Medicine & Medical Sciences, Nelson R. Mandela School of Medicine, College of Health Sciences, University of KwaZulu-Natal, Durban 4001, South Africa; naidoop5@ukzn.ac.za; 3 Division of Research Capacity Development, South African Medical Research Council (SAMRC), Tygerberg, Cape Town 7505, South Africa; 4 Biomedical Sciences Department of Life and Consumer Sciences, College of Agriculture and Environmental Sciences, University of South Africa, Florida Campus, Johannesburg 1710, South Africa

**Keywords:** microRNAs, soil-transmitted helminths, herpes simplex virus type 2/HSV-2, *Nippostrongylus brasiliensis*, co-infection

## Abstract

Soil-transmitted helminths (STHs) and Herpes Simplex Virus type 2 (HSV-2) are highly prevalent infections with overlapping distribution, particularly in resource-poor regions. STH/HSV-2 co-infections may impact female reproductive health. However, many aspects of STH/HSV-2 co-infections, including the role of microRNAs (miRNAs) in regulating female genital tract (FGT) immunity and their potential contribution to pathologies such as chronic inflammation, impaired mucosal defense, and reproductive tract cancers remain unclear. In this study we investigated the miRNA expression profiles in murine FGT tissues following single or co-infection with *Nippostrongylus brasiliensis* (*Nb*) and HSV-2 and explored predicted miRNA-mRNA targets and pathways. An analysis of miRNA sequencing data was conducted to determine differentially expressed (DE) miRNAs between infected FGT tissues and uninfected controls. Ingenuity Pathway Analysis was conducted to predict the immune-related target genes of the DE miRNAs and reveal enriched canonical pathways, top diseases, and biological functions. Selected representative DE miRNAs were validated using RT-qPCR. Our results showed a total of eight DE miRNAs (mmu-miR-218-5p, mmu-miR-449a-5p, mmu-miR-497a-3p, mmu-miR-144-3p, mmu-miR-33-5p, mmu-miR-451a, mmu-miR-194-5p, and mmu-miR-192-5p) in the comparison of *Nb*-infected versus uninfected controls; nine DE miRNAs (mmu-miR-451a, mmu-miR-449a-5p, mmu-miR-144-3p, mmu-miR-376a-3p, mmu-miR-192-5p, mmu-miR-218-5p, mmu-miR-205-3p, mmu-miR-103-3p, and mmu-miR-200b-3p) in the comparison of HSV-2-infected versus uninfected controls; and one DE miRNA (mmu-miR-199a-5p) in the comparison of *Nb*/HSV-2 co-infected versus uninfected controls (*p*-value < 0.05, |logFC| ≥ 1). Core expression analysis showed that, among other canonical pathways, the DE miRNAs and their predicted mRNA targets were involved in neutrophil degranulation, interleukin-4 and interleukin-13 signaling, natural killer cell signaling, interferon alpha/beta signaling, and ISGylation. Additionally, cancer was predicted as one of the significantly enriched diseases, particularly in the co-infected group. This is the first study to provide insights into the FGT miRNA profiles following *Nb* and HSV-2 single and co-infection, as well as the predicted genes and pathways they regulate, which may influence host immunity and pathology. This study highlights the role of miRNAs in regulating FGT immunity and pathology in the context of STH/HSV-2 co-infection.

## 1. Introduction

Soil-transmitted helminths (STHs) and Herpes Simplex Virus type 2 (HSV-2) are widespread infections with overlapping geographical distribution, particularly in resource-poor countries [1,2]. Given their shared geographical distribution, STH/HSV-2 co-infections may impact host immunity and subsequently affect female reproductive health. However, studies investigating STH/HSV-2 co-infections in co-endemic regions remain limited.

Herpes Simplex Virus type 1 (HSV-1) and type 2 (HSV-2) are members of the *Herpesviridae* family, a group of double-stranded DNA viruses capable of establishing lifelong latency. While both HSV-1 and HSV-2 can cause genital herpes, HSV-2 is the primary etiological agent, with an estimated 491.5 million infections reported globally [1,3]. HSV-2 infection, a common sexually transmitted infection, is primarily asymptomatic but may be characterized by recurrent painful genital ulcers. Importantly, HSV-2 infection promotes both susceptibility to and transmission of other sexually transmitted infections, especially HIV [1]. Moreover, HSV-2 has been proposed as a co-factor in the development of cervical cancer, potentially enhancing the oncogenic effects of other agents such as HPV by promoting epithelial disruption and chronic inflammation, impairing local immune responses, and facilitating viral transmission and persistence [4].

Effective viral control requires a robust T-helper type 1 (Th1) immune response, involving innate interferon (IFN)-mediated natural killer cell activity, followed by adaptive immunity via IFN-γ-secreting CD4^+^ and CD8^+^ T cells [5].

STHs, which include roundworms, whipworms, and hookworms, cause over 1.5 billion human infections worldwide. STH infections are most prevalent in tropical and subtropical regions, affecting communities that lack clean water, proper sanitation, and good hygiene practices [2]. STHs typically induce a dominant T-helper type 2 (Th2) immune response and promote immunoregulatory cell populations (Treg or Th3). Both Th2 and Th3 populations suppress Th1 responses. This contrasts with the Th1-induced immunity required for viral control, highlighting the antagonistic nature of the immune responses elicited by STHs and HSV-2. The two arms of the immune system counter-regulate each other such that a predominance of Th2 would downregulate Th1 responses [6,7]. Consequently, STH infections cause systemic immunological changes that influence the outcomes of a range of unrelated co-infections, including important sexually transmitted viral co-infections, such as HIV [8,9,10] and HPV [11,12]. Interestingly, these systemic immunological effects extend to distal uncolonized sites, including the female genital tract (FGT). STHs have been shown to alter female fertility [13] and FGT immunity [12] and may contribute to a tolerant microenvironment that supports the progression of virally induced cancers [14,15]. Other mechanisms include chronic inflammation and immune suppression induced through Th3 regulatory cells, which may negatively affect the immune surveillance of transformed oncogenic cells, thus promoting their uncontrolled proliferation [16].

The question of how STH-induced immune responses influence HSV-2 immune control is less explored but relevant due to the potential immunological interactions between these pathogens. Recently, infection with *Nippostrongylus brasiliensis* (*Nb*), a murine hookworm, was shown to compromise immunity to subsequent genital HSV-2 and exacerbated HSV-2 pathology in the murine FGT [17]. This suggests that STH-induced immune modulation may suppress protective Th1 responses against HSV-2, potentially worsening the course of HSV-2 infection, with serious implications for female sexual and reproductive health. However, many aspects of STH/HSV-2 co-infections remain poorly understood, particularly the role of microRNAs (miRNAs) in mediating host immune responses and pathology. To date, no studies that demonstrate how miRNAs regulate immunity or contribute to disease outcomes in the context of STH/HSV-2 co-infection have been identified.

MiRNAs, which are a class of small, non-coding RNA molecules, are important mediators of gene expression and function. By binding to complementary sequences on target messenger RNAs (mRNAs), miRNAs either inhibit mRNA translation or lead to mRNA degradation [18]. In this way, miRNAs participate in a diverse range of physiological functions, including the regulation of host immune responses. MiRNAs are also involved in disease pathogenesis; dysregulated miRNA expression has been linked to diverse disease contexts, such as infectious diseases, inflammatory diseases, allergies, and cancers. Given their essential multifaceted regulatory roles in physiological and disease processes, miRNAs hold diagnostic and prognostic potential and may serve as therapeutic targets for various diseases [19].

In HSV-2 infections, both host- and HSV-2-derived miRNAs influence HSV-2 infection by regulating viral replication, immune evasion, latency, and various aspects of anti-HSV-2 innate and adaptive immune responses [20,21]. Similarly, in STH infections, host- and helminth-derived miRNAs regulate crucial aspects of antihelminth immunity, influencing intestinal epithelial cell function and the activation of antihelminth innate and adaptive immune cells [22].

Moreover, dysregulated miRNA expression has been implicated in the pathogenesis of various cancers, including those of the female reproductive system. Dysregulation of miRNAs has been linked to cervical, endometrial, and ovarian cancers, influencing key cancer-related pathways such as apoptosis, proliferation, differentiation, and metabolism [23].

The interplay between host-, HSV-2-, and STH-derived miRNAs may profoundly shape the pathogenesis and clinical outcomes of co-infections by modulating immune responses. To better understand STH/HSV-2 co-infections, it is essential to investigate how miRNAs regulate gene expression and immune signaling pathways and how their dysregulation may contribute to chronic inflammation, impaired mucosal immunity, and the progression of FGT-related diseases, including cancer.

In recent years, advances in next-generation sequencing (NGS) platforms and bioinformatics tools have enabled more comprehensive analyses of miRNA functions, interactions, and regulatory networks. NGS offers several advantages compared to traditional methods, such as enhanced sensitivity and higher sequencing depths, which facilitate the identification of numerous host- and pathogen-specific miRNAs [24]. Sequencing approaches have been previously used to characterize HSV-2 [25,26,27,28] and STH miRNAs [29,30,31,32,33]. In the present study, we used an NGS approach to identify differentially expressed (DE) host miRNAs in the FGT tissues of BALB/c mice singly and co-infected with *Nb* and HSV-2. The results of this study will deepen our understanding of the roles of miRNAs in STH/HSV-2 co-infection. Moreover, insights from this work may lead to novel therapeutic miRNA targets and pathways, aimed at mitigating the potential effects of co-infections and improving strategies for infection control.

## 2. Materials and Methods

### 2.1. Ethical Approval

Approval to conduct this study was granted by the Animal Ethics Committee of the University of Cape Town (UCT) (approval number: FHS AEC REF NO: 021_012) and the Animal Research Ethics Committee of the University of KwaZulu Natal (approval number: AREC/00005911/2023).

All experimental procedures were conducted at the Institute of Infectious Diseases and Molecular Medicine, UCT, in compliance with the Section 20 dispensation to conduct animal experiments, which was obtained from the South African Department of Agriculture, Land Reform and Rural Development [reference number: 12/11/1/7/1 (6151KL)]. Only researchers accredited by the South African Veterinary Council performed the experimental procedures.

### 2.2. Experimental Models

#### 2.2.1. Animals

All experiments were conducted as previously described [17]. Female BALB/c mice (Age, 6–10 weeks old; weight, 18–20 g) were bred and maintained under specific pathogen-free conditions at the Research Animal Facility, UCT, South Africa. Mice were group-housed in individually ventilated cages within the same room of the BSL II facility, with no interaction between groups. Food and water were available ad libitum.

Mice were randomly assigned to four experimental groups: (1) singly infected with *Nb*, (2) singly infected with HSV-2, (3) co-infected with *Nb* and HSV-2, and (4) uninfected control groups. Each group comprised 6 mice (*n* = 6).

The estrous cycles of all mice were synchronized by subcutaneously administering 2 mg of Medroxyprogesterone Acetate (Depo Provera^®^) seven days prior to the start of experimental procedures. As pubescent female mice have variable susceptibility to HSV-2 infection, exogenous progesterone was required to synchronize their estrous cycles and reduce experimental variation. Additionally, progesterone modifies the thickness of the vaginal epithelium and the expression of HSV-2 entry receptors, enhancing viral uptake [34].

**Figure 1:**
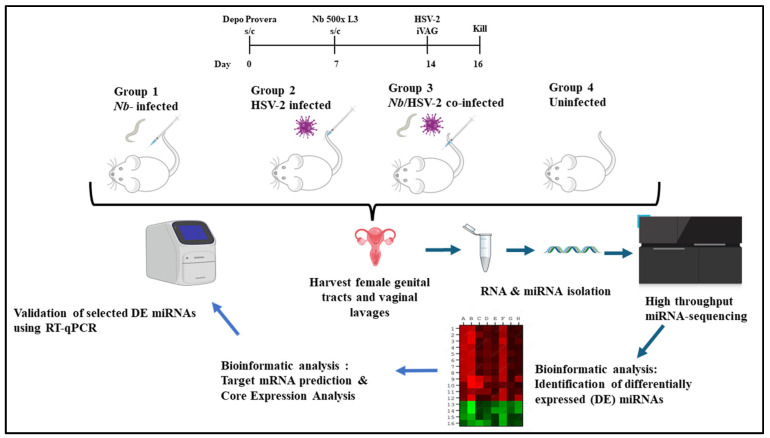
Experimental design: A murine model was used to investigate whether miRNAs in the female genital tract (FGT) are differentially expressed (DE) following acute single or co-infection with *Nippostrongylus brasiliensis* (*Nb*) and HSV-2. Female BALB/c mice aged 6-10-week-old were randomly assigned to four experimental groups (*n* = 6 mice per group): (1) singly infected with *Nb*, (2) singly infected with HSV-2, (3) co-infected with *Nb* and HSV-2, and (4) uninfected control groups. To synchronize the estrous cycles, all mice were given a subcutaneous injection of 2 mg Depo Provera^®^ seven days before the start of experimental procedures. On day 7, mice in Groups 1 and 3 were injected subcutaneously with 500 L3-stage *Nb* larvae. On day 14, i.e., 7 days after *Nb* infection, mice in Groups 2 and 3 were intravaginally challenged with HSV-2. All mice were sacrificed two days after HSV-2 infection. The FGT tissues (excluding ovaries) from each group were isolated and studied comparatively to identify DE miRNAs associated with infection-induced immunity. Target mRNA prediction and core expression analysis were conducted, and a subset of DE miRNAs was randomly selected for validation by RT-qPCR. (Drawn in PPT by author. Free images sourced from https://bioicons.com/ and https://pixabay.com/).

Seven days later, mice in Groups 1 and 3 were injected subcutaneously in the neck scruffs with 500 L3-stage *Nb* larvae. Seven days post-*Nb* infection, mice in Groups 2 and 3 were infected intravaginally with HSV-2 Strain G. All mice were euthanized two days post-HSV-2 infection. The FGT tissues (excluding ovaries) were collected, preserved in RNAlater (ThermoFisher Scientific, Cat. No.: AM7021, Waltham, MA, USA), stored at 4 °C overnight, and then transferred to a −80 °C freezer until further analysis. A schematic overview of the experimental design employed in this study is shown in Figure 1.

#### 2.2.2. *Nb* Maintenance and Infection

*Nb* maintenance and infection were conducted as previously described [17]. Briefly, *Nb* was propagated in male Wistar rats via subcutaneous infection with 5000 L3-stage *Nb* larvae. Thereafter, faecal samples were collected during peak periods of *Nb* egg excretion, which occurs between days 6 and 8 post-infection. To prepare faecal cultures, a faeces/charcoal mix was placed on wet-raised filter paper, and the hatched L3-stage *Nb* larvae that migrated to the edge of the filter paper were collected by gentle washing with water. These L3-stage *Nb* larvae were quantified using a dissecting microscope (Leica M80 Stereo Microscope, Leica Microsystems GmbH, Wetzlar, Germany), followed by resuspension in an appropriate volume of distilled water for use in subsequent infections. Each mouse in Groups 1 and 3 was subcutaneously infected in the scruff of the neck with 500 L3-stage *Nb* larvae suspended in 200 µL of sterile phosphate-buffered saline (PBS) using a 21G needle (Becton, Dickinson & Company, Franklin Lakes, NJ, USA). Infections were administered seven days after Depo-Provera treatment and seven days before viral infection.

#### 2.2.3. Virus

Human herpesvirus 2 strain G (HSV-2, ATCC, VR-734) was cultured in Vero cells at a multiplicity of infection (MOI) of 0.1, as previously described [17]. Two to three days post-infection, both cells and supernatants were harvested, and viral titers were quantified using a plaque assay. Viral stocks were aliquoted and stored at −80 °C until needed.

For HSV-2 infection, mice were anaesthetized via intraperitoneal injection with xylazine (10 mg/kg) and ketamine (100 mg/kg) using a 27 G needle. Each mouse in Groups 2 and 3 was intravaginally challenged with 5 × 10^5^ plaque-forming units (PFU) of HSV-2 strain G in a total volume of 5 μL using a Gilson P10 (Gilson, Inc., Middleton, WI, USA) pipette fitted with a sterile 10 μL filter tip. The pathogen was administered once. Vaginal lavages were collected at two days post-HSV-2 infection. This was performed by washing the vaginal vault 10 times with 50 μL of RNAlater; this washing step was performed three times. To assess the severity of HSV-2-associated illness, mice were monitored by clinical characterization and scored from 0 to 5 as follows: 0 (no pathology), 1 (slight genital/perianal erythema), 2 (genital/perianal swelling and erythema), 3 (genital lesions and/or visible weight loss), 4 (hind limb paralysis and/or purulent lesions), and 5 (premoribund), as previously described [17].

### 2.3. Total RNA Extraction and Quality Control

The murine FGT tissues (excluding ovaries) were isolated, preserved in RNAlater (ThermoFisher Scientific, Cat. No.: AM7021, Waltham, MA, USA), stored at 4 °C overnight, and then transferred to a −80 °C freezer until further analysis. FGT tissues were homogenized in 360 µL RPL buffer (Qiagen, Cat. no./ID. 217684, Hilden, Germany) on ice at 30 s intervals using a handheld homogenizer set on medium speed. Total RNA, including miRNAs, was extracted from FGT tissues using the miRNeasy Tissue/Cells Advanced Micro Kit (Qiagen, Cat. no./ID. 217684, Hilden, Germany), according to the manufacturer’s protocol. Total RNA was eluted in 15 µL of RNAse-free water. RNA concentration and purity were quantified by measuring absorbance at 260 and 280 nm on the NanoDrop 1000 spectrophotometer (Thermo Fisher Scientific, Wilmington, DE, USA). Purified RNA samples were stored at −80 °C.

### 2.4. Library Preparation and Sequencing

Small RNA library preparation and sequencing were conducted using the South African Medical Research Council (SAMRC) Genomics Platform (Cape Town, South Africa). Total RNA was processed using the MGIEasy Small RNA Library Prep Kit (Cat. No.: 940-000196-00, MGI Tech Co., Ltd., Shenzhen, China) and the high-throughput MGI SP-100 sample preparation system, following the manufacturer’s instructions. Briefly, 3′ and 5′ adapters were ligated to small RNAs, followed by reverse transcription and PCR amplification using barcoded primers. Libraries were then subjected to bead-based size selection and purification to enrich for miRNA-sized fragments. Library fragment sizes were assessed using the Agilent 2100 Bioanalyzer (Agilent Technologies, Santa Clara, CA, USA).

Only RNA samples with RNA Integrity Numbers (RIN) ≥ 7 were included for sequencing. A total of 22 samples met this criterion and were distributed across the experimental groups as follows: *Nb*-infected (*n* = 6), HSV-2-infected (*n* = 5), co-infected with *Nb* and HSV-2 (*n* = 5), and uninfected controls (*n* = 6). Sequencing was performed on the DNBSEQ-G400RS platform using single-end 50 bp reads with the Small RNA FCL SE50 sequencing kit (Item No.: 1000019478, MGI Tech Co., Ltd., Shenzhen, China).

### 2.5. Bioinformatics Analysis

#### 2.5.1. Detecting Host-Derived miRNAs and DE miRNAs

**Figure 2:**
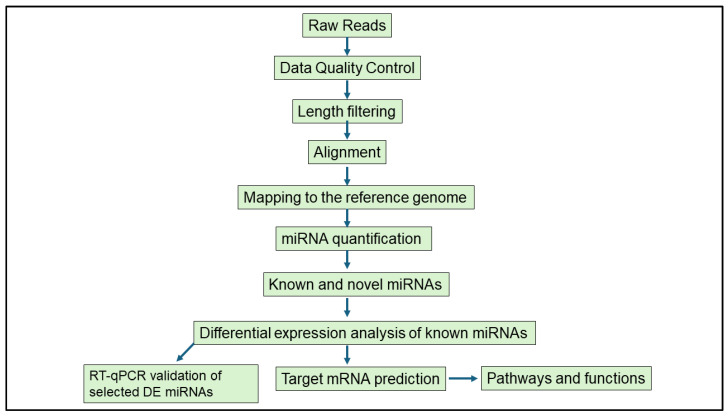
Schematic workflow of the bioinformatics analysis performed to study differentially expressed miRNAs in murine female genital tracts following single and co-infection with *Nippostrongylus brasiliensis* and HSV-2.

Bioinformatics analyses were performed with support from the DIstributed PLatform in OMICS (DIPLOMICS; Cape Town, South Africa). The Comprehensive Analysis Pipeline for miRNA-Sequencing data (CAP-miRSeq) was employed for adapter removal, quality trimming, read alignment, miRNA detection and quantification, and data visualization [35]. The quality of raw sequencing reads was evaluated using FastQC (version 0.12.1), and reads were aligned to the *Mus musculus* reference genome. Known and novel miRNAs were identified and annotated using the miRDeep2 mouse miRNA database. Differential expression analysis was conducted using the edgeR package (version 3.42.4) from Bioconductor (Release 3.17). This model uses empirical Bayes estimation and exact tests based on the negative binomial distribution [35]. DE miRNAs were defined based on a *p*-value < 0.05 and |logFC| ≥ 1, an approach previously used in infection-related miRNA studies [36,37]. The schematic workflow of the miRNA analysis is shown in Figure 2.

#### 2.5.2. MiRNA Target Gene Prediction; Network and Core Expression Analysis

Ingenuity Pathway Analysis (IPA) (Qiagen, Redwood City, CA, USA) was employed to identify the predicted mRNA targets of the DE miRNAs. This was performed using the miRNA target filter tool within IPA, which associates DE miRNAs with their potential target genes by drawing information from data sources such as TargetScan, TarBase, miRecords, and IPA’s knowledge base. These data sources include both experimentally validated miRNA-mRNA interactions and in silico predicted miRNA-mRNA interactions based on literature. This IPA incorporated the logarithmic fold changes and *p*-values of the DE miRNAs. Given that one miRNA may potentially regulate multiple mRNAs [18], to maintain relevance to this study, only mRNA targets involved in immune-related processes/pathways/diseases were analysed. This was performed by filtering all predicted mRNA targets using the following parameters: Source: All; miRNA Confidence Level: Experimentally Validated, High Predicted; Species: Mouse; Pathways: Immune system. To visualize predicted regulatory networks between the DE miRNAs and their mRNA targets, graphs were constructed using the network, overlay and build functions in IPA. Core expression analysis was then performed on all the mRNA targets and the DE miRNAs, with the workflow was set as follows: Core Analysis type: Expression Analysis.

### 2.6. Real-Time Quantitative Polymerase Chain Reaction (RT-qPCR)

A subset of DE miRNAs was randomly selected for validation by RT-qPCR as follows: two miRNAs that were commonly DE in both the *Nb*-infected versus uninfected and HSV-2-infected versus uninfected comparisons, and one unique DE miRNA from each of the single-infection comparisons. In addition, the single DE miRNA identified in the co-infected versus uninfected group was validated. This approach was adopted to verify the miRNA-sequencing results and is consistent with previous studies that explored DE miRNAs in various infection models [37,38,39]. For the first-strand cDNA synthesis, reverse transcription (RT) was conducted using the miRCURY LNA RT Kit (Qiagen, Cat. No. 339340, Hilden, Germany), following the manufacturer’s protocol. Briefly, 200 ng/µL of the extracted RNA was added to the RT reaction and cDNA was made using universal RT primers provided in the kit. All reactions were set up on ice, and aliquots of the cDNA were stored at −20 °C until required. RT-qPCR was performed on an ABI 7000 Real-Time PCR system (Applied Biosystems, Foster City, CA, USA). The miRCURY LNA SYBR Green PCR Kit (Qiagen, Cat. No. 339345, Hilden, Germany) was used in RT-qPCR reactions together with individual miRCURY LNA miRNA PCR (Qiagen, Hilden, Germany) primer assays, following the manufacturer’s protocol. U6 (GeneGlobe ID/Cat. No.: YP02119464|Cat. No.: 339306, Qiagen, Hilden, Germany) was used as an internal control for miRNA template normalization. RT-qPCR reactions were set up for five DE miRNAs using primers—mmu-miR-192-5p (GeneGlobe ID/Cat. No.: YP00204099/339306, Qiagen, Hilden, Germany), mmu-miR-194-5p (GeneGlobe ID/Cat. No.: YP00204080/339306, Qiagen, Hilden, Germany), mmu-miR199a-5p (GeneGlobe ID/Cat. No.: YP00204494/339306, Qiagen, Hilden, Germany), mmu-miR-200b-3p (GeneGlobe ID/Cat. No.: YP00206071/339306, Qiagen, Hilden, Germany), and mmu-miR-218-5p (GeneGlobe ID/Cat. No.: YP00206034/339306, Qiagen, Hilden, Germany). Individual RT-qPCR assays were performed in triplicates with a total reaction volume of 10 μL. The PCR cycling profile was set as follows: 95 °C for 2 min, followed by 40 cycles of denaturation at 95 °C for 10 s, combined annealing/extension at 56 °C for 60 s, and melt curve analysis at 60–95 °C. Data were analysed using the Design and Analysis Software 2.8.0 (Applied Biosystems, Foster City, CA, USA). Expression levels of the miRNAs were measured using CT (threshold cycle). Relative expression levels of miRNAs were calculated by the 2^−ΔΔCt^ method.

### 2.7. Statistical Analysis

Statistical analysis was conducted using Graph Pad Prism software (version 8.0). The significant difference between two groups was analysed by one-way analysis of variance (ANOVA) using Student’s *t*-test. All experimental data for qRT-PCR are expressed as mean ± standard error of mean (SEM). A *p*-value < 0.05 was considered as a significant difference.

## 3. Results

### 3.1. Animal Infection

In this study, we investigated FGT miRNA expression profiles of four different groups: (1) singly infected with *Nb*, (2) singly infected with HSV-2, (3) co-infected with *Nb* and HSV-2, and (4) uninfected control groups. Body weights and pathology scores were assessed in all mice over the course of the study. At day 2 post-HSV-2 infection, no visible signs of disease were detected, and there were no significant differences in body weight or pathology scores among the four groups. Supplementary Figure S1 shows the pathology scores for the infected groups.

### 3.2. Basic Characteristics of Libraries Obtained from miRNA-Sequencing

Of the 24 FGT tissue samples collected from mice across four experimental groups, 22 yielded RNA of sufficient quality (RIN ≥ 7) for miRNA-sequencing. Therefore, the distribution of sequenced samples was as follows: *Nb*-infected (*n* = 6), HSV-2-infected (*n* = 5), *Nb*/HSV-2 co-infected (*n* = 5), and uninfected controls (*n* = 6). Sequencing produced 373,2 million reads that were aligned to the reference genome. Length distribution analysis showed that reads of 21–24 bp were the most abundant. A summary of the numbers of reads aligned to the reference genome, precursor and mature miRNAs, as well as the number of miRNAs reads with ≥5× coverage detected for each sample per group is provided in Supplementary Table S1. A heatmap illustrating the overall trend of miRNA expression across the four experimental groups is shown in Supplementary Figure S2.

### 3.3. Differential Expression of miRNAs

**Figure 3:**
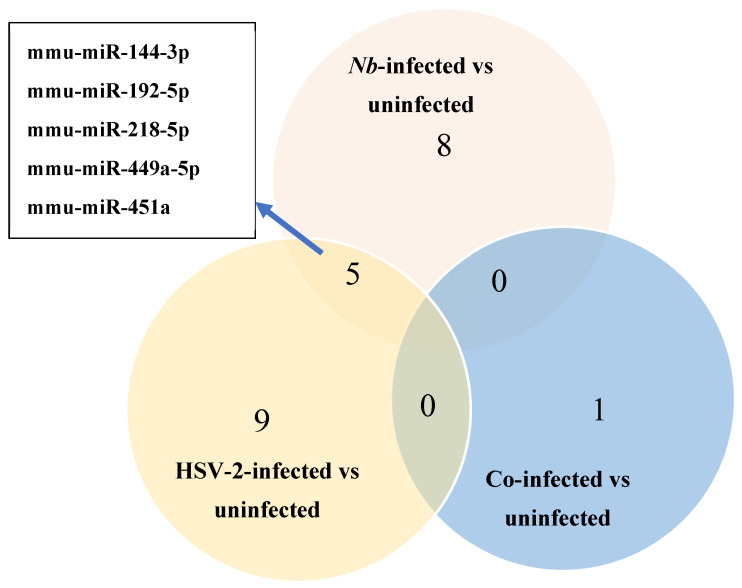
Venn diagram of the number of differentially expressed (DE) miRNAs identified using miRNA-sequencing in the *Nb*-infected versus uninfected, HSV-2-infected versus uninfected, and co-infected versus uninfected comparisons. Five common DE miRNAs were observed in the *Nb*-infected versus uninfected and HSV-2-infected versus uninfected comparisons.

Three comparisons were made between FGT tissues of the infected versus uninfected groups: (1) *Nb*-infected compared to uninfected, (2) HSV-2-infected compared to uninfected, and (3) *Nb*/HSV-2 co-infected compared to uninfected. DE miRNAs in each comparison were identified (*p*-value < 0.05 and |logFC| ≥ 1). We found that within FGT tissues, 8 miRNAs were DE in the *Nb*-infected versus uninfected groups, 9 miRNAs were DE in the HSV-2-infected versus uninfected groups, and 1 miRNA was DE in the *Nb*/HSV-2 co-infected versus uninfected group (Figure 3). The DE miRNAs identified in each comparison are shown in Table 1.

A total of 8 DE miRNAs were identified in the *Nb*-infected compared to uninfected FGT tissues, of which 6 miRNAs were upregulated (mmu-miR-218-5p, mmu-miR-449a-5p, mmu-miR-497a-3p, mmu-miR-144-3p, mmu-miR-33-5p, and mmu-miR-451a) and 2 were downregulated (mmu-miR-194-5p and mmu-miR-192-5p). A total of 9 DE miRNAs were identified when the HSV-2-infected and uninfected FGT tissues were compared, of which 4 miRNAs were upregulated (mmu-miR-451a, mmu-miR-449a-5p, mmu-miR-144-3p, and mmu-miR-376a-3p) and 5 miRNAs (mmu-miR-192-5p, mmu-miR-218-5p, mmu-miR-205-3p, mmu-miR-103-3p, and mmu-miR-200b-3p) were downregulated. In the comparison between the co-infected and uninfected FGT tissues, only 1 DE miRNA (mmu-miR-199a-5p) was identified and was downregulated. We observed 5 common DE miRNAs in the *Nb*-infected versus uninfected and HSV-2-infected versus uninfected comparisons (mmu-miR-144-3p, mmu-miR-192-5p, mmu-miR-218-5p, mmu-miR-449a-5p, and mmu-miR-451a). Among these, mmu-miR-218-5p was upregulated in the *Nb*-infected versus uninfected comparison but downregulated in the HSV-2-infected versus uninfected comparison.

### 3.4. MiRNA Target Gene Prediction, Network and Core Expression Analysis

**Figure 4:**
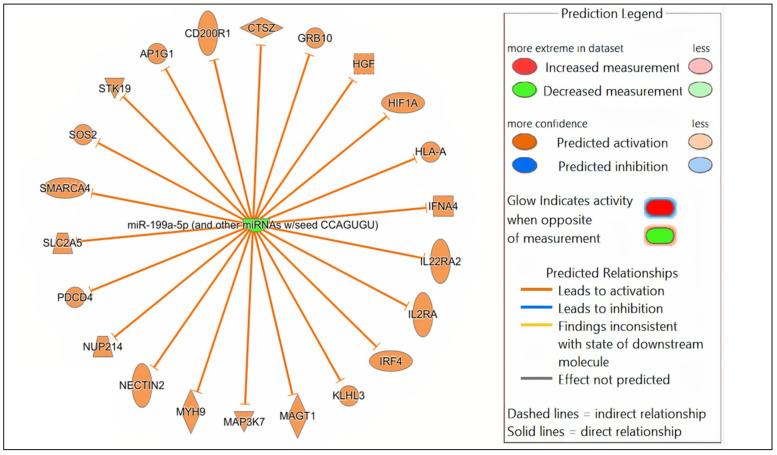
Network analysis. Representative graph showing the differentially expressed (DE) miRNAs-mRNA network in *Nb*/HSV-2 co-infection. The DE miRNA and the predicted targets and functional relation are represented as nodes and lines. The colour of the nodes represents their predicted expression status: orange (predicted activation) and blue (predicted inhibition). The different shapes of the nodes represent the nature of the molecules based on their activity, e.g., cytokines, enzymes, etc.

Potential mRNA targets of all the DE miRNAs in each comparison were predicted by Qiagen IPA. In brief, the DE miRNAs for each comparison were individually submitted to IPA. All predicted targets identified in IPA, and the corresponding DE miRNAs, were included in the subsequent core expression analysis. Multiple predicted mRNA target genes with known roles in host immunity were identified. Networks were constructed in IPA to visualize the predicted regulatory functions of the DE miRNAs (Figure 4, Supplementary Figures S3 and S4). A summary of the predicted targets of the DE miRNAs for each comparison is shown in Table 2, Table 3 and Table 4.

All the DE miRNAs and their predicted targets for each group-wise comparison were used in IPA for the subsequent core expression analysis. For each group-wise comparison, target genes of upregulated and downregulated miRNAs were collectively analysed.

In the comparison of *Nb*-infected versus uninfected FGT tissues, immune-related canonical pathways were significantly enriched (*p*-value < 0.05). “Neutrophil degranulation” was the canonical pathway with the highest significance. Other immune-related canonical pathways, including “interleukin-4 and interleukin-13 signaling”, were also significantly enriched. Among the top diseases and biological functions, “inflammatory response” (diseases and disorders), “cell-to-cell signaling and interaction” (molecular and cellular function), and “haematological system development and function” (physiological system development and function) were identified.

In the comparison of HSV-2-infected versus uninfected FGT tissues, “role of macrophages, fibroblasts and endothelial cells in rheumatoid arthritis” was the canonical pathway with the highest significance, followed by “neutrophil degranulation”, and “hepatitis B chronic liver pathogenesis signaling pathway”. Among the top diseases and biological functions, “inflammatory response” (diseases and disorders), “cell death and survival” (molecular and cellular function), and “haematological system development and function” (physiological system development and function) were identified.

**Figure 5:**
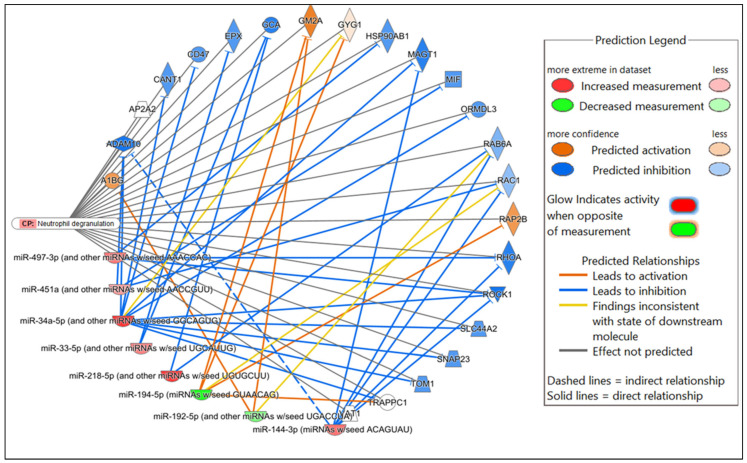
Network displaying miRNA-predicted mRNA targets involved in the neutrophil degranulation canonical pathway in the comparison of *Nb*-infected versus uninfected FGT tissues.

**Figure 6:**
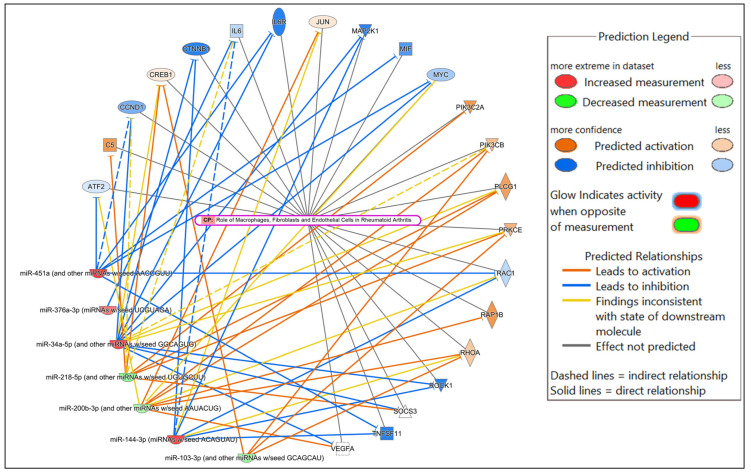
Network displaying miRNA-predicted mRNA targets involved in regulating the role of macrophages, fibroblasts and endothelial cells in rheumatoid arthritis canonical pathway in the comparison of HSV-2-infected versus uninfected FGT tissues.

**Figure 7:**
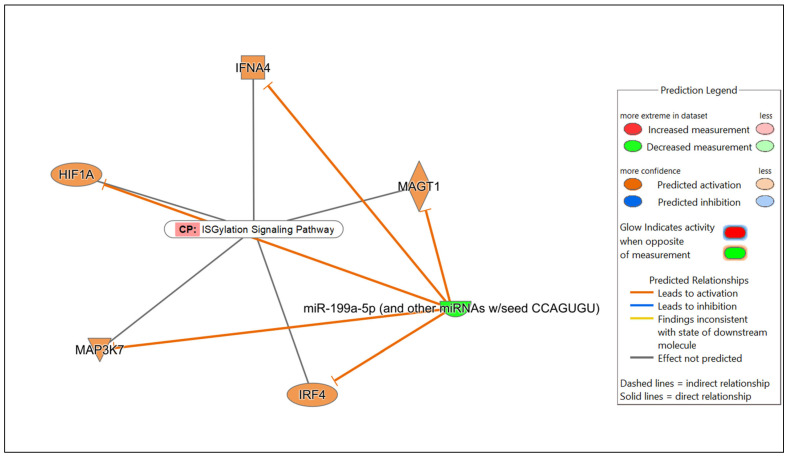
Network displaying miRNA-predicted mRNA targets involved in regulating the ISGylation signaling pathway in the comparison of *Nb*/HSV-2 co-infected versus uninfected FGT tissues.

In the comparison of *Nb*/HSV-2 co-infected versus uninfected FGT tissues, the most significant canonical pathway was the “ISGylation signaling pathway”. Immune-related canonical pathways including “natural killer cell signaling”, and “interferon alpha/beta signaling” were also significantly enriched. Among the top diseases and biological functions, “cancer” (diseases and disorders), “cell-to-cell signaling and interaction” (cellular and molecular function), and “haematological system development and function” (physiological system development and function) were identified. The top 5 canonical pathways, diseases, molecular and cellular functions, and physiological system development and functions of each comparison are shown in Table 5, Table 6 and Table 7. Networks of the top canonical pathway identified in each comparison are shown in Figure 5, Figure 6 and Figure 7.

### 3.5. Confirmation of DE miRNAs Using Real-Time Quantitative Polymerase Chain Reaction (RT-qPCR)

**Figure 8:**
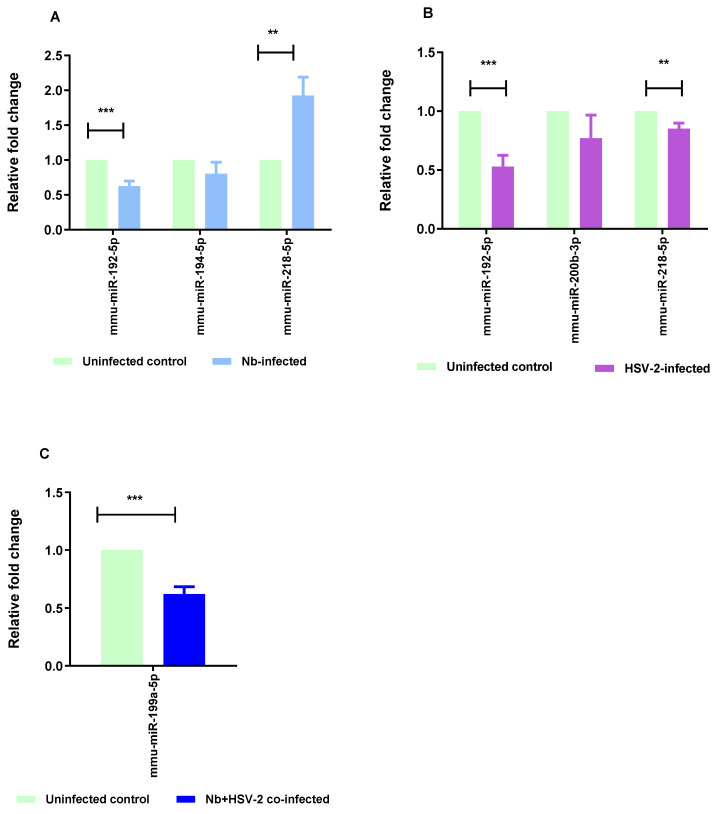
RT-qPCR validation. RT-qPCR was performed to validate the results of the miRNA-sequencing. Five miRNAs, representative of differentially expressed miRNAs from single *Nb* and HSV-2 infections and co-infection, (mmu-miR-192-5p, mmu-miR-194-5p, mmu-miR-218-5p, mmu-miR-200b-3p, and mmu-miR-199a-5p) were selected for validation. The following comparisons are shown: (**A**) *Nb*-infected versus uninfected, (**B**) HSV-2-infected versus uninfected, and (**C**) *Nb*/HSV-2 co-infected versus uninfected. The relative expression levels were normalized to U6, which was used as the internal control. The data are expressed as mean ± SEM, *** *p* < 0.001; ** *p* < 0.01.

The RT-qPCR method was used to validate five selected DE miRNAs, comprising two common to both the *Nb*- and HSV-2-infected groups, one unique to each of the single infections, and the single DE miRNA in the co-infected group. These included mmu-miR-192-5p, mmu-miR-194-5p, mmu-miR-218-5p, mmu-miR-200b-3p, and mmu-miR-199a-5p. The RT-qPCR results exhibited a consistent trend in expression changes with those observed in the miRNA sequencing results, with significant results when comparing mmu-miR-218-5p (*p*-value = 0.0061) and mmu-miR-192-5p (*p*-value = 0.0004) in the *Nb*-infected versus uninfected groups. Significant values were also observed when comparing mmu-miR-218-5p (*p*-value = 0.0100) and mmu-miR-192-5p (*p*-value = 0.0006) in the HSV-2-infected versus uninfected groups, and mmu-miR-199a-5p (*p*-value = 0.0001) in the *Nb*/HSV-2 co-infected versus uninfected groups (Figure 8).

## 4. Discussion

In this study, we investigated miRNA expression profiles in the murine FGT following single and co-infection with *Nb* and HSV-2. Differential expression analysis of miRNA-sequencing data revealed 8 DE miRNAs in *Nb*-infected, 9 in HSV-2-infected, and 1 in co-infected tissues compared to uninfected controls (*p* < 0.05, |logFC| ≥ 1). IPA predicted immune-related target genes and identified enrichment in pathways such as neutrophil degranulation, IL-4 and IL-13 signaling, natural killer cell signaling, interferon alpha/beta signaling, and ISGylation. Cancer was also identified as a significantly enriched disease category, particularly in the co-infected group. Selected miRNAs were validated by RT-qPCR, supporting the reliability of the sequencing data. A detailed discussion of the immune- and pathology-associated DE miRNAs and predicted pathways identified in our study follows below.

### 4.1. Immune-Related DE miRNAs and Pathways

In this study, we investigated FGT miRNA expression profiles of four different groups: (1) singly infected with *Nb*, (2) singly infected with HSV-2, (3) co-infected with *Nb* and HSV-2, and (4) uninfected control groups. *Nb*, which is closely related to human hookworm, induces a transient, self-limiting infection characterized by a strong Th2 immune response. Clinical symptoms are mild and short-lived, with the infection typically resolving within 10–13 days [40]. In contrast, intravaginal HSV-2 infection in female mice, results in a localized infection that typically produces visible symptoms between days 3 and 5 post-infection [17,41]. In our study, the absence of overt clinical symptoms such as significant weight loss and pathology at day 2 post-HSV-2 infection is consistent with previous studies, which indicate that early-stage HSV-2 infection in murine models presents with minimal clinical manifestations [17].

We used NGS to characterize the miRNA profiles of FGT tissues of BALB/c mice singly infected with either *Nb* or HSV-2 or co-infected with both pathogens. We further identified the predicted target mRNAs of DE miRNAs and associated canonical pathways, diseases, and biological functions using IPA. Previous studies have only reported on the immunoregulatory roles of miRNAs during single infection with STHs [42,43,44,45,46] or HSV-2 [27,47,48], thus highlighting the critical role of miRNAs in regulating host cellular changes and immune responses during either infection. To our knowledge, this is the first study to examine miRNA expression profiles within murine FGT tissues following *Nb*/HSV-2 co-infection.

In this study, we demonstrated that miRNA expression profiles in the FGT were dysregulated following single infections with *Nb* or HSV2, as well as *Nb*/HSV-2 co-infection. Specifically, we observed both differences and similarities in the DE miRNA signatures based on the type of infection. During single *Nb* infection, 8 miRNAs (mmu-miR-194-5p, mmu-miR-218-5p, mmu-miR-449a-5p, mmu-miR-192-5p, mmu-miR-497a-3p, mmu-miR-144-3p, mmu-miR-33-5p, and mmu-miR-451a) were DE when compared to the uninfected control group. During single HSV-2 infection, 9 miRNAs (mmu-miR-192-5p, mmu-miR-451a, mmu-miR-449a-5p, mmu-miR-218-5p, mmu-miR-144-3p, mmu-miR-376a-3p, mmu-miR-205-3p, mmu-miR-103-3p, and mmu-miR-200b-3p) were DE when compared to the uninfected control group. We also observed 5 common DE miRNAs (mmu-miR-144-3p, mmu-miR-192-5p, mmu-miR-218-5p, mmu-miR-449a-5p, and mmu-miR-451a) during single *Nb* infection and single HSV-2 infection. Interestingly, among the common DE miRNAs, only miR-218-5p showed opposite expression patterns in response to single *Nb* infection and single HSV-2 infection.

Although most of the DE miRNAs identified in our study have not yet been described in the context of *Nb* or HSV-2 infections, we identified several miRNAs with established roles in immune responses and inflammation. Firstly, among the common DE upregulated miRNAs, miR-144-3p modulated immune responses to bacterial and viral infections by influencing processes such as autophagy, lipid metabolism, cytokine and chemokine production, and inflammation [49,50,51,52]. Upregulated levels of miR-449a-5p in the female reproductive tract have been reported in previous studies [53,54,55]. In other studies, miR-449a-5p was shown to regulate inflammatory responses in recipient T cells and promote apoptosis through the AKT signaling pathway [56,57]. Studies have reported that miR-451a plays a role in immune responses by modulation of neutrophil chemotaxis and macrophage M2 polarization [58,59]. Upregulated levels of miR-451a have been reported in sepsis and in response to both Gram-positive and Gram-negative microorganisms [60,61,62]. Previously, it was reported that miR-192-5p alleviated asthma-induced inflammation in a murine model. Its overexpression was associated with decreased levels of ovalbumin-specific IgE, IL-4, IL-5, and IL-13, contributing to improved asthma outcomes by limiting airway remodeling and autophagy through the downregulation of matrix metalloproteinase (MMP)-16 and autophagy related 7 (ATG7) [63]. Similarly, upregulation of miR-192-5p was observed in serum exosomes of individuals with non-alcoholic fatty liver disease (NAFLD), where it was essential for activation of pro-inflammatory macrophages and NAFLD progression through the regulation of the Rictor/Akt/FoxO1 signaling pathway [64]. Serum miR-192-5p expression was significantly linked to various hepatitis B virus (HBV) infection marker levels and was identified as a biomarker for pegylated-IFN efficacy in chronic HBV treatment, thereby suggesting its role in HBV replication and antiviral immunity [65]. In a murine model of *Schistosoma japonicum* infection, miR-192-5p was highly abundant in the plasma extracellular vesicles of infected mice compared to uninfected mice [66]. In contrast, we observed a downregulation of miR-192-5p in both *Nb*-infected and HSV-2-infected FGT tissues and the predicted activation of several immune-related genes, including *A1BG*, *DHX58*, *GM2A*, *NLRC5*, *RSAD2*, *STK3*, and *ZEB1*. Among these, *ZEB1* has been described during helminth infection, where TLR9 stimulation increased *ZEB1* expression in cDC1 dendritic cells. *ZEB1* depletion led to reduced activation and production of IL-6, IL-10, and IL-12 and a shift in CD4^+^ T helper cells toward a Th2 phenotype [67]. The role of *ZEB1* has been studied in HSV-1 infection; the viral protein *ICP0* promoted *ZEB1* and *ZEB2* degradation, resulting in an increased expression of the miR-183 cluster [68]. In Epstein–Barr Virus (EBV) infection, interaction of *ZEB1* with the ZV element regulated the transition between latency and lytic replication [69]. Therefore, in our study, the downregulation of miR-192-5p and its regulation of genes, such as *ZEB1*, may reflect an important component of innate immune responses during *Nb* and HSV-2 infections which requires further investigation.

In addition, we identified miRNAs known to modulate the NF-κB and Toll-Like Receptor (TLR) signaling pathways (e.g., mmu-miR-194-5p, mmu-miR-218-5p, mmu-miR-200b-3p). Previously it was reported that miR-194-5p exerted an indirect effect on NF-κB by targeting *TRIM23* and *C21ORF91*, genes which are required for NF-κB induction, thereby mitigating inflammation [70]. Increased expression of miR-194-5p was associated with decreased *TRAF6* levels and suppressed TNF-α production in palmitic acid-activated THP-1 monocytes, suggesting a negative regulation of the TLR4 signaling pathway [71]. Additionally, miR-194-5p directly targeted the *TLR4* gene, regulating cytokine production in response to *Salmonella* infection [72]. In this study, we observed the downregulation of miR-194-5p and the predicted activation of its immune-related genes, including *FASLG*, *GYG1*, *IL9*, *RAP2B*, *SUMO2*, and *TAB3.* Among these, *IL9* is a characteristic Th2 cytokine produced during innate immunity and known to confer protection against helminth infections [73,74,75], including *Nb* [76].

Similarly, the upregulation of miR-218-5p was inversely correlated with immune defense and inflammatory responses, NF-κB pathway modulation, antigen processing and presentation, and chemokine signaling [77]. In the present study, we observed an upregulation of miR-218-5p during single *Nb* infection. This finding is particularly interesting within the broader context of STH-induced immune modulation, as STHs, including *Nb*, are known to stimulate robust Th2 responses and immunomodulatory mechanisms that suppress pro-inflammatory Th1 immune pathways [6]. Our observation of upregulation of miR-218-5p, together with the predicted downregulation of its immune-related mRNA targets, suggests a potential role for this miRNA in mediating the immunomodulatory effects associated with *Nb* and warrants further investigation into its specific regulatory functions during *Nb*-associated immune responses. In contrast, we found that miR-218-5p was downregulated during single HSV-2 infection. Our findings are consistent with previous studies, which have demonstrated a role for miR-218-5p in viral infections. For example, in porcine reproductive and respiratory syndrome virus (PRRSV), miR-218 downregulation facilitated viral replication through inhibition of type I IFN responses [78]. In porcine epidemic diarrhoea virus (PEDV), inhibition of heterogeneous nuclear ribonucleoprotein A3 (HNRNPA3) expression through miR-218-5p enhanced cellular lipid accumulation and viral replication [79].

Studies have shown that miR-200b-3p regulates viral replication. Our observation of decreased miR-200b-3p in the HSV-2-infected FGT tissues compared to uninfected controls is consistent with previous viral studies. For example, miR-200b-3p suppressed IFN-I production, driven by NF-κB and IRF3-mediated pathways, by directly targeting the *TBK1* gene. Its inhibition was associated with increased IFN-I production and broad-spectrum antiviral effects [80]. The miR-200 family, including miR-200b-3p, targeted *ZEB1* and *ZEB2*, suppressing the *BZLF1* gene and promoting EBV reactivation [81]. Decreased miR-200b-3p levels were associated with cytopathic inflammation during human cytomegalovirus (HCMV) infection, suggesting a role in the host response to viral pathogens [82].

Appreciable changes during co-infection were not observed in this study, since only 1 DE miRNA was identified when the co-infected group was compared to the uninfected group. Interestingly, miR-199a-5p was significantly downregulated when the co-infected group was compared to the uninfected group. The role of miR-199a-5p has not been previously investigated in co-infections. However, previous studies have shown that the upregulation of miR-199a-5p facilitated hepatitis C virus (HCV) replication via the PI3K/Akt, Ras/ERK and Wnt/β-catenin pathways [83]. Following HSV-1 infection, miR-199a-5p and miR-199a-3p suppressed *ARHGAP21*, thereby regulating *Cdc42* and impairing the secondary envelopment of HSV-1, demonstrating their antiviral activity [84]. miR-199a-5p-deficient mice showed enhanced inflammatory cell infiltration, pro-inflammatory cytokine expression and impaired intestinal barrier function following dextran sulphate sodium (DSS)-induced colitis, suggesting the anti-inflammatory role of miR-199a [85]. In helminth infections, downregulated levels of miR-199a-5p were reported in *Trichinella spiralis* infection [86]. Given the known antiviral and anti-inflammatory functions of miR-199a-5p, its downregulation in the co-infected FGT tissues observed in our study, may contribute to the enhanced vaginal pathology previously reported in *Nb*/HSV-2 co-infected mice [17]. Decreased levels of miR-199a-5p may potentially impair the host’s ability to effectively regulate infection-associated inflammation associated with *Nb*/HSV-2 co-infection; however, the mechanisms by which this may occur requires further investigation.

Notably, the detection of only a single DE miRNA in the co-infected group, in contrast to several identified in the *Nb* and HSV-2 single infections, potentially reflects both biological and technical influences. Biologically, we hypothesize that the opposing immune responses induced by each pathogen (Th2-mediated polarization by *Nb* and a Th1-mediated immune response by HSV-2) may counteract one another, leading to a dampened immune state and limited miRNA dysregulation. This masking effect could substantially reduce the number of DE miRNAs identified, even though biologically meaningful regulatory dynamics are at play. In support, one compelling observation was the opposing expression of miR-218-5p in single *Nb* and HSV-2 infections. Moreover, according to IPA, miR-218-5p is predicted to regulate (i.e., either activate or inhibit) its immune-related target genes depending on its direction of expression. Such opposing regulation could lead to a net expression normalization during co-infection. However, technical factors must also be acknowledged, including small sample size, biological variability, and the analysis of acute infection at a single time point, all of which may limit the detection of subtle miRNA expression changes.

Taken together, our findings are suggestive of a complex interplay between *Nb* and HSV-2 and highlight the need for more integrated and functional analyses to fully characterize regulatory mechanisms during co-infection.

In support, computational analysis also revealed that the predicted target genes and DE miRNAs were statistically enriched in biological functions, including those associated with innate immunity. This implies that these miRNAs may contribute to critical FGT mucosal defenses by mobilizing innate immune responses during infection [87]. Core expression analysis of the DE miRNAs and their predicted targets has also identified the statistical over-representation of several canonical pathways previously implicated in the host response to infection, such as neutrophil degranulation, interleukin-4 and interleukin-13 signaling, interferon alpha/beta signaling, and ISGylation signaling pathways. For example, neutrophils play a protective role in helminth infections by limiting parasite survival and spread, mainly through the formation of neutrophil extracellular traps (NETs) and neutrophil degranulation. Neutrophils are the first cells to traffic infection sites, influencing the Th2 immune response by attracting other immune cells and priming macrophages toward an M2 phenotype [88,89,90,91]. Interleukin (IL)-4 and IL-13 are essential in Th2 immunity, enhancing resistance to helminths and neutralizing toxins by promoting Th2 and T follicular helper cell differentiation, IgE production, expansion of basophils and eosinophils, mast cell activation, M2 macrophage polarization, and goblet cell hyperplasia [92]. For example, IL-4 and IL-13 produced by innate immune cells were sufficient to promote effective Th2 immunity against *Nb* [93]. ISGylation, which is a post-translational modification involving the conjugation of ISG15, is induced by type I IFNs and plays an essential role in innate antimicrobial defense and immune regulation [94].

### 4.2. Pathology-Related DE miRNAs and Pathways

MiRNAs modulate key cellular processes, including proliferation, apoptosis, differentiation, and metabolism, that are frequently dysregulated in cancer. In female reproductive cancers, altered miRNA expression has been associated with ovarian, cervical, endometrial, and vulvar cancers. Depending on context and target genes, miRNAs may function as oncogenes or tumor suppressors [23].

In our study, cancer was predicted as one of the significantly enriched diseases, most notably in the co-infected group. This enrichment potentially reflects infection-induced miRNA dysregulation of immune- and inflammation-related signaling pathways commonly associated with carcinogenesis. Potential roles in cancer for some of the DE miRNAs observed in our study can be speculated based on previous literature and the results of our analyses. For example, several miRNAs identified in our study, including miR-451a, miR-449a-5p, miR-218-5p, miR-144-3p, miR-376a-3p, miR-199a-5p, miR-205-3p, miR-103-3p, and miR-200b-3p, have been previously implicated in female reproductive cancers [23]. Several are known tumor suppressors, including miR-192-5p [95,96], miR-194-5p [97], miR-451a [98], miR-218-5p [99], miR-144-3p [100], miR-199a-5p [101,102], miR-200b-3p [103], miR-205-3p [104], and miR-449a-5p [105,106], with dysregulation linked to enhanced proliferation, invasion, and poor prognosis in cervical, ovarian, or endometrial cancers. In contrast, miR-376a-3p was significantly elevated in ovarian cancer and associated with the clinical stages of disease [107], while miR-103-3p was associated with oncogenic roles in cervical cancer by promoting tumor cell survival [108].

In the context of our study, the DE miRNAs observed in the *Nb*-infected group suggests that STH-induced immune modulation may influence host miRNA networks in ways that overlap with cancer-related signaling. The concurrent downregulation of key tumor-suppressive miRNAs (miR-194-5p, miR-192-5p) and upregulation of others (miR-218-5p, miR-449a-5p miR-497a-3p, miR-144-3p, and miR-451a) could reflect *Nb*-induced immune modulation in the FGT. This immune modulation may influence miRNA expression in a context-dependent manner, potentially contributing to a microenvironment that supports malignant transformation [14,15].

Similarly, in the HSV-2-infected group, the downregulation of key tumor suppressors (miR-192-5p, miR-218-5p, miR-205-3p, and miR-200b-3p) may impair epithelial repair and promote a microenvironment favourable to carcinogenesis, while upregulated miRNAs (miR-451a, miR-449a-5p, and miR-144-3p) may reflect antiviral or tissue-protective responses.

Importantly, in the co-infected group, the significant downregulation of miR-199a-5p may reflect an immunological interaction between *Nb* and HSV-2, which contribute to pathology and epithelial stress. As highlighted earlier, considering the established anti-viral, anti-inflammatory, and tumor-suppressive roles of miR-199a-5p, its reduced expression in co-infected FGT tissues may play a role in the increased vaginal pathology previously reported in *Nb*/HSV-2 co-infected mice [17]. It is also plausible that the loss of miR-199a-5p in this context may favour an increased risk for malignant transformation, highlighting the need for further mechanistic studies.

Taken together, our study provides evidence that FGT miRNA expression profiles during single and co-infection with *Nb* and HSV-2 are altered, and that these changes could influence the regulation of host immunity to either pathogen, while potentially overlapping with pathology-related pathways, such as cancer. While our core expression analysis suggests a link between miRNA regulation and cancer pathways during single, and more notably during co-infection, these findings remain speculative and require mechanistic validation.

## 5. Limitations of the Study and Future Work

While the murine model of *Nb* and/or HSV-2 infection replicates only certain aspects of human infection [6,109], our findings highlight the significant role of miRNA regulation and expression in single and co-infection settings. These insights may have broader implications for host immunity and pathogenesis for humans. Notably, our study provides evidence that miRNAs influence host immune responses and key biological pathways during single and co-infection with *Nb* and HSV-2. Despite the strengths of this work, including (a) an NGS approach and (b) single infection and co-infection, it has some limitations. The main limitation is the limited sample size. This limitation was addressed by confirming the miRNA expression of selected miRNAs using RT-qPCR and specific primers in a validation study. In addition, biological variation between individual mice could have confounded some of our observations. We also acknowledge that a comprehensive validation of all DE miRNAs is ideal to strengthen the reliability of the miRNA-sequencing results. However, due to resource constraints, it was not feasible to validate the full complement of DE miRNA identified through miRNA-sequencing. While the consistent expression trends observed in the subset of validated miRNAs using RT-qPCR support our miRNA-sequencing data, we acknowledge the need to expand the validation of DE miRNAs and their target genes and to confirm their mechanistic roles in our future work. Additionally, while our core expression analysis suggests a potential link between miRNA regulation and cancer-related pathways, these preliminary findings remain speculative and require validation through future mechanistic studies. Therefore, although our findings are insightful in the context of STH/HSV-2 co-infection, they should be validated through larger-scale investigations. Future research should include comprehensive analyses of miRNA expression profiles in response to each infection separately and in combination to better understand pathogen-specific miRNA expression changes. In addition, research on the impact of acute versus chronic infection on miRNA expression profiles and the prognostic potential of miRNAs is required. Though these were not within the scope of our current study, our present findings form a basis for such future studies. Our future studies will focus on validating target gene expression using RNA-sequencing and evaluation of the mechanistic roles of DE miRNAs using in vitro and in vivo models. Elucidation of the mechanisms by which DE miRNAs regulate host immunity could lead to novel approaches to control or treat STHs and HSV-2 infections in co-endemic settings.

## 6. Conclusions

In summary, this study demonstrates that single and co-infection with *Nb* and HSV-2 are characterized by different miRNA expression patterns, which may underlie differences in host immune responses and infection-induced pathologies. Several miRNAs were found to be significantly dysregulated in both single and co-infection settings, including some not previously reported in co-infection models. Predicted target genes were associated with immune functions, and core expression analysis revealed strong associations with biological and immunological processes. In addition, the affected pathways also intersect with those implicated in important diseases, such as cancer. Overall, our findings suggest that the DE miRNAs identified in this study play central roles in immunity in the setting of single and co-infection with *Nb* and HSV-2, underscoring the importance of miRNA-focused research in understanding host immunity to and potential pathology associated with STH/HSV-2 co-infection.

## Figures and Tables

**Table 1:** Differentially expressed microRNAs that were identified in each comparison.

*Nb*-Infected Versus Uninfected			
**Name of miRNA**	Log Fold Change (LogFC) *	*p*-Value *	**Up/Downregulation**
mmu-miR-194-5p	−3.27	8.00 × 10^−4^	Down
mmu-miR-218-5p	2.09	4.20 × 10^−3^	Up
mmu-miR-449a-5p	2.17	4.30 × 10^−3^	Up
mmu-miR-192-5p	−1.92	8.38 × 10^−3^	Down
mmu-miR-497a-3p	1.43	1.57 × 10^−2^	Up
mmu-miR-144-3p	1.51	2.65 × 10^−2^	Up
mmu-miR-33-5p	1.34	4.05 × 10^−2^	Up
mmu-miR-451a	1.06	4.44 × 10^−2^	Up
**HSV-2-infected versus Uninfected**			
**Name of miRNA**	Log Fold Change (LogFC) *	*p*-Value *	**Up/Downregulation**
mmu-miR-192-5p	−2.94	3.48 × 10^−4^	Down
mmu-miR-451a	1.88	1.37 × 10^−3^	Up
mmu-miR-449a-5p	1.72	2.88 × 10^−3^	Up
mmu-miR-218-5p	−1.54	9.12 × 10^−3^	Down
mmu-miR-144-3p	1.71	9.73 × 10^−3^	Up
mmu-miR-376a-3p	1.26	1.05 × 10^−2^	Up
mmu-miR-205-3p	−1.46	1.91 × 10^−2^	Down
mmu-miR-103-3p	−1.31	2.07 × 10^−2^	Down
mmu-miR-200b-3p	−1.17	3.77 × 10^−2^	Down
***Nb*** **/HSV-2 co-infected versus Uninfected**			
**Name of miRNA**	Log Fold Change (LogFC) *	*p*-Value *	**Up/Downregulation**
mmu-miR-199a-5p	−2.46	4.88 × 10^−2^	Down

Footnote: * DE miRNAs were identified based on *p*-Value < 0.05 and |logFC| ≥ 1.

**Table 2:** *Nb*-infected versus uninfected: list of predicted immune-related targets for the differentially expressed miRNAs.

Name of miRNA	Predicted Immune-Related Targets	Predicted Regulatory Effect
mmu-miR-144-3p	*AP1S3*, *CTNNB1*, *EP300*, *HIF1A*, *ITCH*, *JUN*, *MAGT1*, *MAP3K8*, *MEF2A*, *MTOR*, *RAC1*, *RHOA*, *ROCK1*, *TNFSF11*, *UBE2D1*, *UBE2D3*	Predicted inhibition
mmu-miR-192-5p	*A1BG*, *DHX58*, *GM2A*, *NLRC5*, *RSAD2*, *STK3*, *ZEB1*	Predicted activation
mmu-miR-194-5p	*FASLG*, *GYG1*, *IL9*, *RAP2B*, *SUMO2*, *TAB3*	Predicted activation
mmu-miR-218-5p	*C5*, *CD200R1*, *COL1A1*, *DDX41*, *LMO7*, *NUP50*, *PIK3C2A*, *PLCG1*, *RAB6A*, *RICTOR*, *RNF41*, *RPS6KA3*, *SH3KBP1*, *SOCS3*, *SPSB1*, *UBE2H*, *VAMP7*, *VAT1*	Predicted inhibition
mmu-miR-33-5p	*EPX3*, *GCA*	Predicted inhibition
mmu-miR-449a-5p	*ADAM10*, *AP2A2*, *BCL2*, *BCL6*, *BTN1A1*, *C9*, *CANT1*, *CASP2*, *CCL22*, *CCND1*, *CD47*, *CD79A*, *CREB1*, *CSF1R*, *CTNNB1*, *DEFB124*, *DNM1L*, *FBX017*, *FCER1A*, *GRAP2*, *GSTO1*, *IL23R*, *IL6R*, *ISG20*, *MAP2K1*, *MAP3K3*, *MAPT*, *MUC5B*, *MYC*, *MYH9*, *NECTIN2*, *ORMDL3*, *PTPN4*, *RNF4*, *ROCK1*, *SIAH1*, *SLC44A2*, *SMAD3*, *SNAP23*, *TOM1*, *TP53* *, *TRAPPC1*, *TRIM21*, *TXN*, *UBE2L3*, *VAMP2*, *VAT1*, *VEGFA*, *WASF1*	Predicted inhibition * Predicted activation (*TP53*)
mmu-miR-451a	*ATF2*, *EDAR*, *MIF*, *RAC1*, *WASF1*, *YBX1*	Predicted inhibition
mmu-miR-497a-3p	*HSP90AB1*, *IL19*	Predicted inhibition

**Table 3:** HSV-2-infected versus uninfected: list of predicted immune-related targets for the differentially expressed miRNAs.

Name of miRNA	Predicted Immune-Related Targets	Predicted Regulatory Effect
mmu-miR-103-3p	*ARIH1*, *BTLA*, *CRKL*, *EEF2*, *FBXW11*, *ICOS*, *IL10RB*, *N4BP1*, *NUP58*, *PIK3CB*, *PRKCE*, *PTGS2*, *RAB10*, *SDCBP*	Predicted activation
mmu-miR-144-3p	*AP1S3*, *CTNNB1*, *EP300*, *HIF1A*, *ITCH*, *JUN*, *MAGT1*, *MAP3K8*, *MEF2A*, *MTOR*, *RAC1*, *RHOA*, *ROCK1*, *TNFSF11*, *UBE2D1*, *UBE2D3*	Predicted inhibition
mmu-miR-192-5p	*A1BG*, *DHX58*, *GM2A*, *NLRC5*, *RSAD2*, *STK3*, *ZEB1*	Predicted activation
mmu-miR-200b-3p	*AP1S2*, *ARIH1*, *CRKL*, *ELOC*, *MSN*, *PLCG1*, *PTEN*, *PTPN12*, *PTPN13*, *RAP1B*, *SEC23A*, *SNAP25*, *UBE2W*, *ZEB1*	Predicted activation
mmu-miR-205-3p	*PSMA5*	Predicted activation
mmu-miR-218-5p	*C5*, *CD200R1*, *COL1A1*, *DDX41*, *LMO7*, *NUP50*, *PIK3C2A*, *PLCG1*, *PRKG1*, *RAB6A*, *RICTOR*, *RNF41*, *RPS6KA3*, *SH3KBP1*, *SOCS3*, *SPSB1*, *UBE2H*, *VAT1*	Predicted activation
mmu-miR-449a-5p	*ADAM10*, *AP2A2*, *BCL2*, *BCL6*, *BTN1A1*, *C9*, *CANT1*, *CASP2*, *CCL22*, *CCND1*, *CD47*, *CD79A*, *CREB1*, *CSF1R*, *CTNNB1*, *DEFB124*, *DNM1L*, *FBXO17*, *FCER1A*, *GRAP2*, *GSTO1*, *IL23R*, *IL6R*, *ISG20*, *MAP2K1*, *MAP3K3*, *MAPT*, *MUC5B*, *MYC*, *MYH9*, *NECTIN2*, *ORMDL3*, *PTPN4*, *RNF4*, *ROCK1*, *SIAH1*, *SLC44A2*, *SMAD3*, *SNAP23*, *TOM1*, *TP53* *, *TRAPPC1*, *TRIM21*, *TXN*, *UBE2L3*, *VAMP2*, *VAT1*, *VEGFA*, *WASF1*	Predicted inhibition * Predicted activation (*TP53*)
mmu-miR-376a-3p	*CTSO*, *IL6*, *KIF5A*, *TRIM9*	Predicted inhibition
mmu-miR-451a	*ATF2*, *EDAR*, *MIF*, *RAC1*, *WASF1*, *YBX1*	Predicted inhibition

**Table 4:** *Nb*/HSV-2 co-infected versus uninfected: list of predicted immune-related targets for the differentially expressed miRNAs.

Name of miRNA	Predicted Immune-Related Targets	Predicted Regulatory Effect
mmu-miR-199a-5p	*AP1G1*, *CD200R1*, *CTSZ*, *GRB10*, *HGF*, *HIF1A*, *HLA-A*, *IFNA4*, *IL22RA2*, *IL2RA*, *IRF4*, *KLHL3*, *MAGT1*, *MAP3K7*, *MYH9*, *NECTIN2*, *NUP214*, *PDCD4*, *SLC2A5*, *SMARCA4*, *SOS2*, *STK19*	Predicted activation

**Table 5:** *Nb*-infected versus uninfected: summary of core expression analysis.

Top Canonical Pathways		
Name	*p*-Value *	Overlap †
Neutrophil degranulation	1.60 × 10^−18^	4.8% (23/477)
Role of macrophages, fibroblasts, and endothelial cells in rheumatoid arthritis	1.16 × 10^−16^	5.6% (19/337)
Hypoxia signaling in the cardiovascular system	3.49 × 10^−16^	15.4% (12/78)
Interleukin-4 and interleukin-13 signaling	7.80 × 10^−16^	11.7% (13/111)
NGF Signaling	2.47 × 10^−15^	10.7% (13/121)
**Top Diseases and Biological Functions**		
**Diseases and disorders**		
**Name**	***p*** -Value Range **	**No. of Molecules ‡**
Inflammatory Response	1.85 × 10^−8^–2.05 × 10^−36^	87
Infectious Diseases	2.83 × 10^−8^–1.73 × 10^−29^	76
Organismal Injury and Abnormalities	3.24 × 10^−8^–1.73 × 10^−29^	107
Cancer	3.24 × 10^−8^–9.38 × 10^−21^	89
Haematological Disease	3.10 × 10^−8^–2.56 × 10^−20^	62
**Molecular and Cellular Functions**		
**Name**	***p*** -Value Range **	**No. of Molecules ‡**
Cell-To-Cell Signaling and Interaction	2.03 × 10^−8^–3.36 × 10^−29^	74
Cell Death and Survival	3.13 × 10^−8^–1.61 × 10^−28^	77
Cellular Development	3.24 × 10^−8^–3.51 × 10^−27^	80
Cellular Growth and Proliferation	3.24 × 10^−8^–3.51 × 10^−27^	81
Cellular Movement	3.31 × 10^−8^–3.05 × 10^−26^	70
**Physiological System Development and Function**		
**Name**	***p*** -Value Range **	**No. of Molecules ‡**
Haematological System Development and Function	3.15 × 10^−8^–2.36 × 10^−32^	80
Tissue Morphology	3.15 × 10^−8^–2.36 × 10^−32^	68
Haematopoiesis	2.06 × 10^−8^–3.51 × 10^−27^	47
Lymphoid Tissue Structure and Development	3.15 × 10^−8^–3.51 × 10^−27^	60
Tissue Development	3.24 × 10^−8^–3.51 × 10^−27^	66

Footnote: * Indicates how statistically significant the enrichment is for a particular pathway/function. Calculated using the right-tailed Fisher’s exact test; ** Range of significant *p*-values for the diseases/functions grouped in a specific category; † Refers to the number of genes from the input dataset that are found in a pathway/function compared to the total number of genes known to be involved in that pathway/function; ‡ Refers to the count of molecules (genes/proteins) from the input dataset that are associated with a specific pathway/disease/function.

**Table 6:** HSV-2-infected versus uninfected: summary of core expression analysis.

Top Canonical Pathways		
Name	*p*-Value *	Overlap †
Role of Macrophages, Fibroblasts, and Endothelial Cells in Rheumatoid Arthritis	8.68 × 10^−19^	6.5% (22/337)
Neutrophil degranulation	7.97 × 10^−18^	5.0% (24/477)
Hepatitis B Chronic Liver Pathogenesis Signaling Pathway	4.28 × 10^−17^	9.0% (17/188)
Hypoxia Signaling in the Cardiovascular System	6.96 × 10^−17^	16.7% (13/78)
Hepatic Fibrosis Signaling Pathway	7.52 × 10^−17^	5.3% (22/416)
**Top Diseases and Biological Functions**		
**Diseases and disorders**		
**Name**	***p*** -Value Range **	**No. of Molecules ‡**
Inflammatory Response	4.64 × 10^−9^–4.75 × 10^−37^	100
Infectious Diseases	8.67 × 10^−10^–1.66 × 10^−31^	86
Organismal Injury and Abnormalities	5.03 × 10^−9^–1.66 × 10^−31^	127
Cancer	5.03 × 10^−9^–5.40 × 10^−22^	108
Tumor Morphology	1.99 × 10^−9^–3.40 × 10^−20^	38
**Molecular and Cellular Functions**		
**Name**	***p*** -Value Range **	**No. of Molecules ‡**
Cell Death and Survival	4.92 × 10^−9^–4.28 × 10^−30^	89
Cell-To-Cell Signaling and Interaction	4.51 × 10^−9^–1.61 × 10^−28^	85
Cellular Movement	4.64 × 10^−9^–4.39 × 10^−28^	82
Cellular Development	5.00 × 10^−9^–2.13 × 10^−26^	99
Cellular Growth and Proliferation	5.00 × 10^−9^–2.13 × 10^−26^	98
**Physiological System Development and Function**		
**Name**	***p*** -Value Range **	**No. of Molecules ‡**
Haematological System Development and Function	4.64 × 10^−9^–1.17 × 10^−35^	90
Tissue Morphology	3.75 × 10^−9^–1.17 × 10^−35^	80
Immune Cell Trafficking	4.64 × 10^−9^–2.46 × 10^−27^	66
Haematopoiesis	1.06 × 10^−9^–2.13 × 10^−26^	53
Lymphoid Tissue Structure and Development	3.75 × 10^−9^–2.13 × 10^−26^	70

Footnote: * Indicates how statistically significant the enrichment is for a particular pathway/function. Calculated using the right-tailed Fisher’s exact test; ** Range of significant *p*-values for the diseases/functions grouped in a specific category; † Refers to the number of genes from the input dataset that are found in a pathway/function compared to the total number of genes known to be involved in that pathway/function; ‡ Refers to the count of molecules (genes/proteins) from the input dataset that are associated with a specific pathway/disease/function.

**Table 7:** *Nb*/HSV-2 co-infected versus uninfected: summary of core expression analysis.

Top Canonical Pathways		
Name	*p*-Value *	Overlap †
ISGylation Signaling Pathway	3.25 × 10^−8^	4.6% (5/109)
Glucocorticoid Receptor Signaling	4.96 × 10^−7^	1.2% (7/600)
Hepatitis B Chronic Liver Pathogenesis Signaling Pathway	1.89 × 10^−5^	2.1% (4/188)
Natural Killer Cell Signaling	2.55 × 10^−5^	2.0% (4/203)
Interferon alpha/beta Signaling	3.75 × 10^−5^	3.9% (3/76)
**Top Diseases and Biological Functions**		
**Diseases and disorders**		
**Name**	***p*** -Value Range **	**No. of Molecules ‡**
Cancer	5.15 × 10^−3^–5.25 × 10^−13^	21
Haematological Disease	5.15 × 10^−3^–5.25 × 10^−13^	17
Immunological Disease	4.90 × 10^−3^–5.25 × 10^−13^	18
Organismal Injury and Abnormalities	5.15 × 10^−3^–5.25 × 10^−13^	23
Inflammatory Response	5.15 × 10^−3^–3.81 × 10^−10^	20
**Molecular and Cellular Functions**		
**Name**	***p*** -Value Range **	**No. of Molecules ‡**
Cell-To-Cell Signaling and Interaction	5.15 × 10^−3^–3.81 × 10^−10^	14
Cellular Development	5.15 × 10^−3^–6.04 × 10^−10^	20
Cellular Growth and Proliferation	5.15 × 10^−3^–6.04 × 10^−10^	20
Cellular Function and Maintenance	5.15 × 10^−3^–4.91 × 10^−8^	16
Cellular Movement	5.15 × 10^−3^–6.64 × 10^−8^	15
**Physiological System Development and Function**		
**Name**	***p*** -Value Range **	**No. of Molecules ‡**
Haematological System Development and Function	5.15 × 10^−3^–3.81 × 10^−10^	18
Immune Cell Trafficking	4.29 × 10^−3^–3.81 × 10^−10^	14
Lymphoid Tissue Structure and Development	5.15 × 10^−3^–3.63 × 10^−9^	15
Tissue Morphology	4.94 × 10^−3^–5.17 × 10^−9^	16
Haematopoiesis	5.15 × 10^−3^–3.17 × 10^−7^	10

Footnote: * Indicates how statistically significant the enrichment is for a particular pathway/function. Calculated using the right-tailed Fisher’s exact test; ** Range of significant *p*-values for the diseases/functions grouped in a specific category; † Refers to the number of genes from the input dataset that are found in a pathway/function compared to the total number of genes known to be involved in that pathway/function; ‡ Refers to the count of molecules (genes/proteins) from the input dataset that are associated with a specific pathway/disease/function.

## Data Availability

The original contributions presented in this study are included in the article/Supplementary Material. Further inquiries can be directed to the corresponding author.

## Supplementary Materials

The following supporting information can be downloaded at https://www.mdpi.com/article/10.3390/microorganisms13081734/s1, Supplementary Figure S1: Pathology scoring was assessed between the groups post-infection; Supplementary Table S1: Summary of the numbers of reads, precursor and mature miRNAs, and the number of miRNAs reads with ≥5x coverage detected for each sample in groups: A (Uninfected controls), B (singly infected with *Nb*), C (singly infected with HSV-2), and D (*Nb*/HSV-2 co-infected); Supplementary Figure S2: Heat map illustrating the overall trend of miRNA expression changes across the four groups: Samples A3-8 (Uninfected control), B1-6 (*Nb*-infected), C2-6 (HSV-2-infected), and D1,3-6 (*Nb*/HSV-2 co-infected); Supplementary Figure S3: Network showing predicted miRNA-target mRNA relationships in the comparison of *Nb*-infected versus uninfected controls; Supplementary Figure S4: Network showing predicted miRNA-target mRNA relationships in the comparison of HSV-2-infected versus uninfected controls.
