# Supplementary material for: Characterization of microRNA Expression Profiles of Murine Female Genital Tracts Following Nippostrongylus brasiliensis and Herpes Simplex Virus Type 2 Co-Infection
Source: Microorganisms. 2025 Jul 24;13(8):1734. doi: 10.3390/microorganisms13081734 (PMC12388830; doi:10.3390/microorganisms13081734)
Supplement: Supplementary file 1 [file microorganisms-13-01734-s001.zip › microorganisms-3717928-supplementary.pdf]

SUPPLEMENTARY DATA

RESEARCH ARTICLE

Characterization of microRNA expression profiles of murine female genital tracts following *Nippostrongylus brasiliensis* and Herpes Simplex Virus type 2 co-infection

Roxanne Pillay [1,2,3], Pragalathan Naidoo [2,3] and Zilungile L. Mkhize-Kwitshana [3,4*]

[1] Department of Biomedical Sciences, Faculty of Applied and Health Sciences, Mangosuthu University of Technology, Umlazi, Durban 4031, South Africa
[2] Department of Medical Microbiology, College of Health Sciences, School of Laboratory Medicine & Medical Sciences, Nelson R. Mandela School of Medicine, University of KwaZulu-Natal, Durban 4001, South Africa
[3] Division of Research Capacity Development, South African Medical Research Council (SAMRC), Tygerberg, Cape Town 7505, South Africa
[4] Biomedical Sciences Department of Life and Consumer Sciences, College of Agriculture and Environmental Sciences, University of South Africa, Florida Campus, Johannesburg 1710, South Africa
* Correspondence: mkhizzl@unisa.ac.za

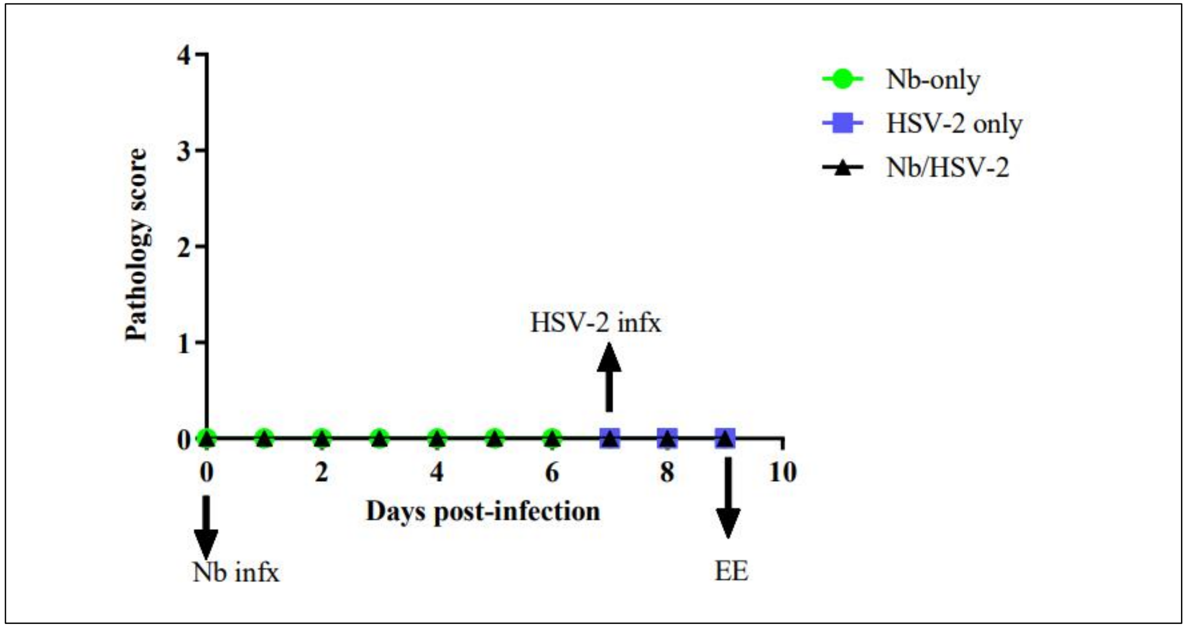

Supplementary Figure S1: Pathology scoring was assessed between the groups post-infection.

Footnote: *Nb*: *Nippostrongylus brasiliensis*, HSV-2: Herpes Simplex Virus type 2, Infx: Infection, EE: Experimental Endpoint

Supplementary Table S1: Summary of the numbers of reads, precursor and mature miRNAs, and the number of miRNAs reads with ≥5x coverage detected for each sample in groups: A (Uninfected controls), B (singly infected with *Nb*), C (singly infected with HSV-2), and D (*Nb*/HSV-2 co-infected)

Sample	Reads sent to Aligner	Precursor miRNA Reads	Mature miRNA Reads	Known miRNA with ≥ 5x coverage
A3	20119581	16688	13507171	741
A4	22080150	4137	17162131	521
A5	43263780	28420	31877037	881
A6	22526233	6702	19214322	562
A7	14224394	8600	8186252	702
A8	4884676	2800	3063711	532
B1	19624390	13593	11968979	746
B2	17030994	11229	11707727	753
B3	11648074	4165	9199198	511
B4	11567618	3844	8804494	521
B5	13966191	8533	9236569	745
B6	12537759	11634	7241392	639
C2	17625373	12412	10987299	712
C3	15080880	11177	9437592	727
C4	19293332	4796	16064770	482
C5	13878779	8558	10324506	678
C6	19629940	10223	13664854	725
D1	18003779	3660	15570168	497
D3	20211339	7282	13381540	518
D4	14516633	13575	7602598	690
D5	13704205	9591	8545108	673
D6	7818418	3668	5984503	467

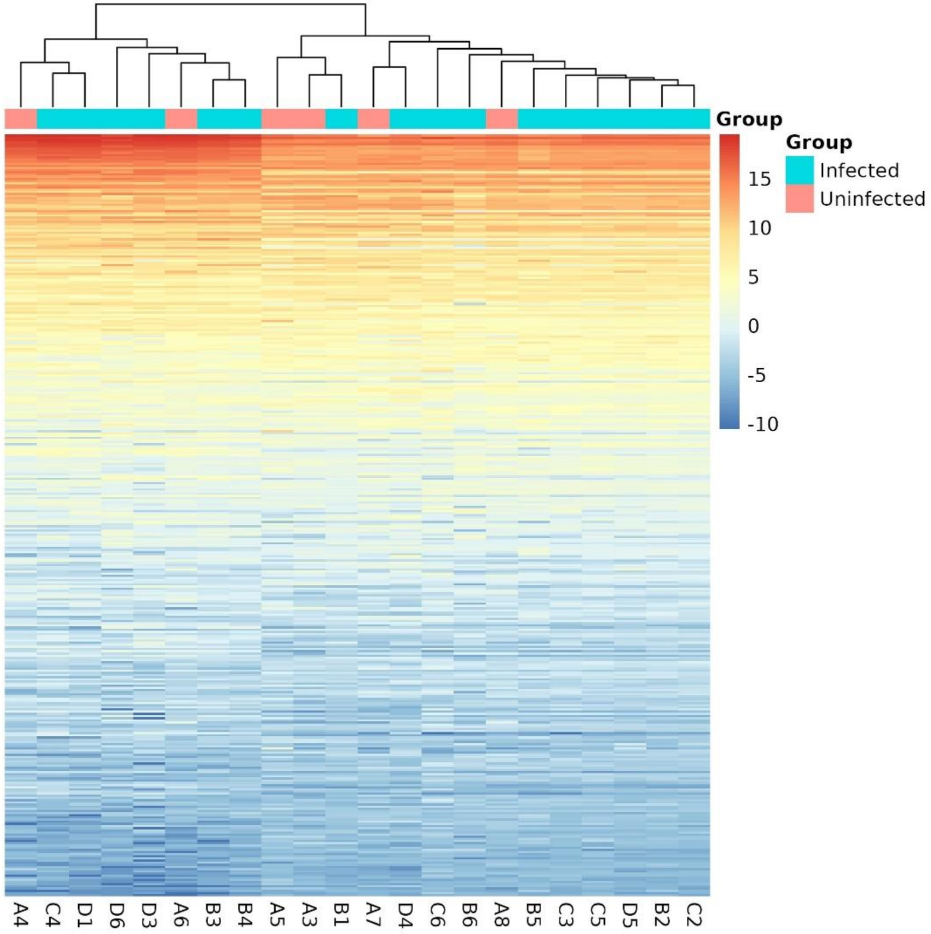

Supplementary Figure S2: Heat map illustrating the overall trend of miRNA expression changes across the four groups: Samples A3-8 (Uninfected control), B1-6 (*Nb*-infected), C2-6 (HSV-2-infected), and D1,3-6 (*Nb*/HSV-2 co-infected)

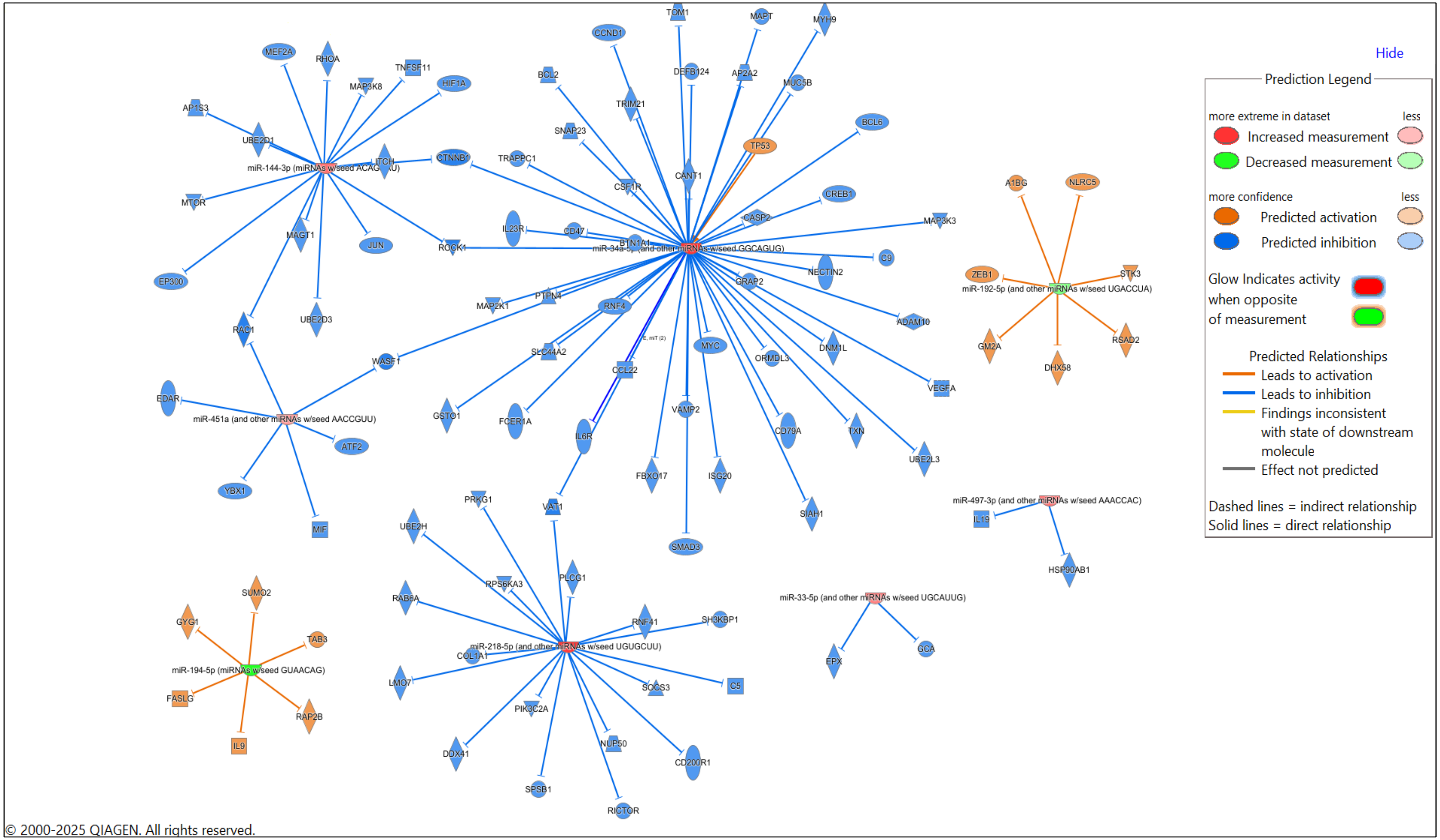

© 2000-2025 QIAGEN. All rights reserved.

Supplementary Figure S3: Network showing predicted miRNA-target mRNA relationships in the comparison of *Nb*-infected versus uninfected controls.

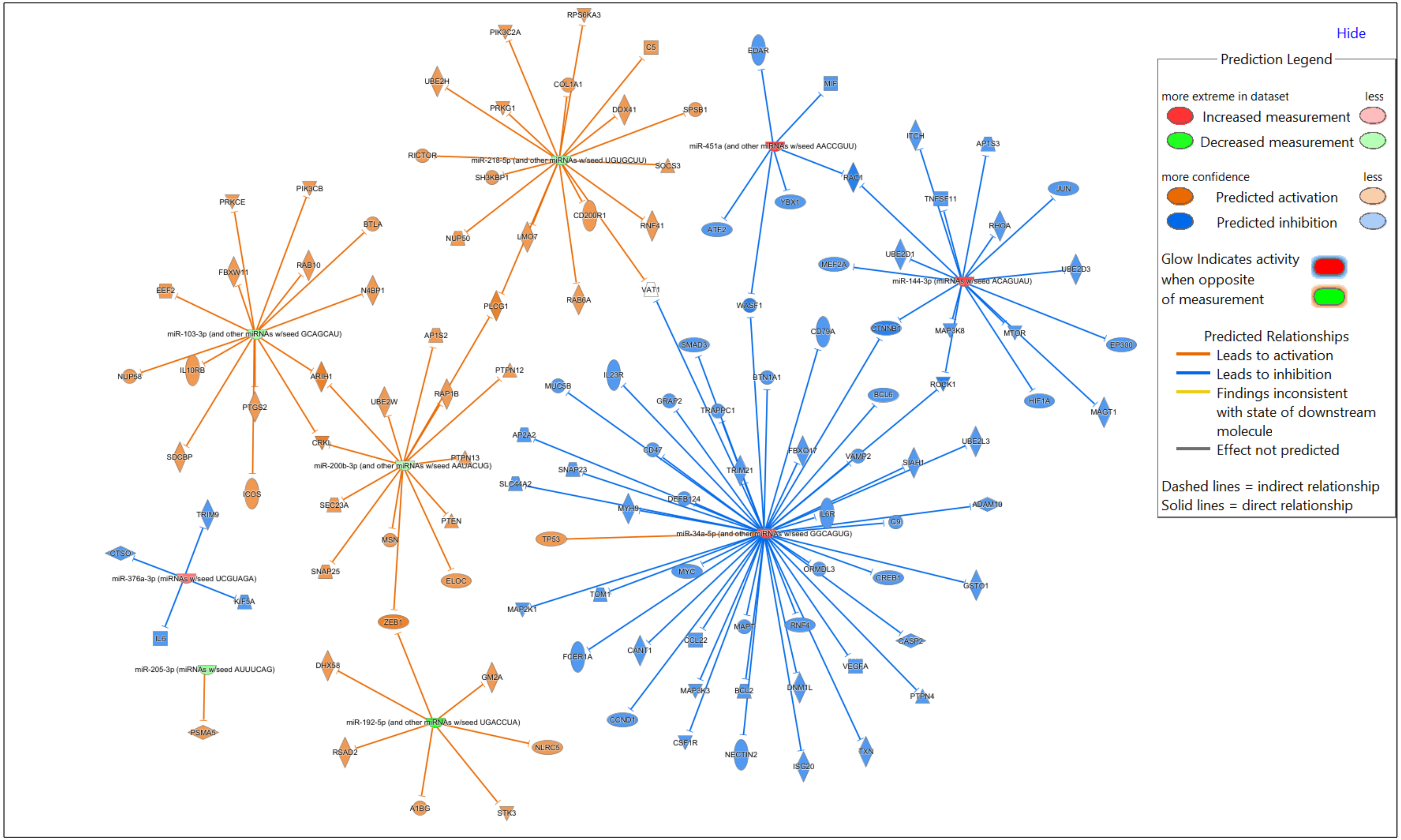

Supplementary Figure S4: Network showing predicted miRNA-target mRNA relationships in the comparison of HSV-2-infected versus uninfected controls.
